# Keeping kids safe for active travel to school: A mixed method examination of school policies and practices and children’s school travel behaviour

**DOI:** 10.1016/j.tbs.2020.05.008

**Published:** 2020-10

**Authors:** Erika Ikeda, Suzanne Mavoa, Alana Cavadino, Penelope Carroll, Erica Hinckson, Karen Witten, Melody Smith

**Affiliations:** a School of Sport and Recreation, Auckland University of Technology, Auckland, New Zealand; b Centre for Diet & Activity Research (CEDAR), MRC Epidemiology Unit, School of Clinical Medicine, University of Cambridge, Cambridge, UK; c Melbourne School of Population and Global Health, University of Melbourne, Melbourne, Australia; d SHORE and Whāriki Research Centre, Massey University, Auckland, New Zealand; e School of Population Health, The University of Auckland, Auckland, New Zealand; f School of Nursing, The University of Auckland, Auckland, New Zealand

**Keywords:** School travel behaviour, Active travel, School policy, Safety, Traffic, Mixed methods

## Abstract

Active school travel contributes to children’s physical, mental and social wellbeing. The prevalence of children’s active school travel, however, has been declining in many developed countries. Gaining insights into school culture and environments in relation to school travel behaviour is crucial to inform interventions. Using a multiphase mixed methods approach, this study aimed to provide a comprehensive understanding of how school policies and practices supported or inhibited school travel behaviour in Auckland, New Zealand. Data were drawn from Neighbourhoods for Active Kids, a cross-sectional study of 1085 children aged 8–13 years between February 2015 and December 2016. School representatives were interviewed regarding their policies and practices related to school travel behaviour and traffic around school, and the data were analysed thematically. An overarching theme, sub-themes and categories were contextualised for quantitative modelling using objectively measured school variables (school socioeconomic status, active school travel programme, built environments around school). Mixed effects multinomial logistic regression models were employed to determine associations between school travel mode and objectively measured child (sociodemographic characteristics, traffic safety perceptions) and school variables. Safety was the core concept of school travel policies, procedures and programmes. Significant differences in child variables, school socioeconomic status, and cycle lanes and traffic lights around school were found between children who actively travelled or used public transport to school and those driven to school. Overall, this study demonstrated the important role of school policy and procedures and the potential application of an intersectoral approach for interventions to support changes in school travel behaviour.

## Highlights

• School travel policy and practices predominantly focus on road traffic safety.
• Support from local government was the anchor of school travel policies and practices.
• Safe pedestrian crossings around school may facilitate active school travel.
• Biking and public transport to school was inequitable by school socioeconomic status.
• An intersectoral approach is needed to support active school travel.

## Graphical abstract

**Figure:**
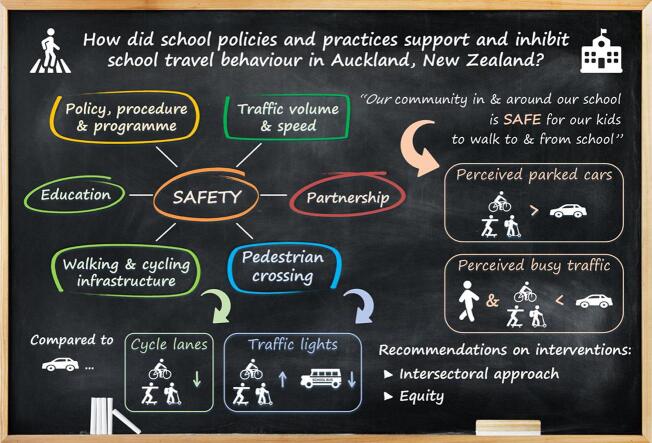

## 1 Introduction

Childhood physical inactivity underlies a number of non-communicable diseases (e.g., heart disease, stroke, some cancers, diabetes), and has given rise to a global phenomenon of child obesity and overweight (World Health Organization, 2018). According to the 2018 Global Matrix 3.0 Physical Activity Report Card, the average percentage of children and youth who met recommendations on physical activity for health (i.e., at least 60 min of daily moderate-to-vigorous intensity physical activity) ranged from 27 to 33% (Aubert et al., 2018).

Active travel (e.g., walking and cycling from place to place) is a key way of accumulating physical activity on a daily basis (Faulkner et al., 2009, Larouche et al., 2014b), and improving physical, mental and social wellbeing (Ikeda et al., 2018a, Larouche et al., 2014b, Sun et al., 2015, Van Dijk et al., 2014). Mode shifts from passive (i.e., motorised) travel to active travel can also contribute to environmental sustainability and economic benefits (Grabow et al., 2012, Hosking et al., 2011, Lindsay et al., 2011, Woodcock et al., 2009, Zapata-Diomedi et al., 2017). Despite these benefits, a low prevalence of active travel in children and youth and a car-centric culture has been an issue particularly in North America, UK, Australia, New Zealand and other developed countries (Aubert et al., 2018, Sattlegger and Rau, 2016). Over the last five years, however, a plateau (e.g., New Zealand, South Africa) or slight improvement (e.g., USA, Australia) in active travel has been observed. One potential contribution to this status is the rapid growth of programmes to promote active travel particularly focusing on school travel behaviour such as Safe Routes to School, School Travel Plans, and walking school buses (Aubert et al., 2018, Ikeda et al, in press, Larouche et al., 2018, Tremblay et al., 2016, Tremblay et al., 2014).

New Zealand, with a total population of just under five million in 2019 (Statistics New Zealand, 2019), has sprawling urban development and high car dependency (Leung et al., 2017). The New Zealand Health Survey 2016/17 showed less than half (45%) of children and youth ages 5–14 years usually actively travelled to school (Ministry of Health, 2017). Those children and youth who usually travel actively are more likely to be male and older children (ages 10–14 years) than female, younger children or older adolescents (Smith et al., 2018). Traditionally, active school travel has been higher in children and youth living in areas of higher deprivation; however, recent data suggest this pattern may be shifting (Ikeda et al., 2018a, Ministry of Health, 2014, Ministry of Health, 2015, Ministry of Health, 2017). Research that identifies factors associated with active school travel and evaluates existing policies and interventions for active school travel will be indispensable to build knowledge of ‘what works for whom in what context’ (Larouche et al., 2018).

A complex mechanism of associations between school travel behaviour and multiple factors at individual, social, environmental and policy levels has been thoroughly documented (Ikeda et al., in press). Ikeda and colleagues (2019) developed the Children’s School Travel Behaviour Model (C-STBM) and demonstrated direct and indirect (mediated) relationships between school travel behaviour and six domains (i.e., built environment, social environment, household characteristics, household beliefs, child characteristics, child beliefs) in the context of Auckland, New Zealand. With the exception of household characteristics, active school travel was significantly associated with one or more variables from the five domains (i.e., objectively-measured distance to school; neighbourhood social environment; household beliefs about traffic safety, social interaction and convenience; child age and sex; child beliefs of neighbourhood safety, and independent mobility) (Ikeda et al., 2019). Despite acknowledging the important role of the school environment (i.e., policy and practices) in the C-STBM, the previous study omitted this seventh domain due to an insufficient sample size of schools (n = 19) in order to perform multilevel analyses in structural equation modelling (Ikeda et al., 2019). This study, therefore, continues to test the C-STBM particularly focusing on the domain of the school environment associated with school travel behaviour.

A school acts as a facilitator of interventions for active school travel by providing safe and supportive environments, and as a key driver of reinforcing active school travel habits and values (Buttazzoni et al., 2018, Crawford and Garrard, 2013, Hawley et al., 2019). Love and colleagues (2019) evaluated three interventions for school travel behaviour and independent mobility in Australian children (i.e., TravelSmart, Ride to School, Safe Routes to School). Social capital (i.e., social connectedness) of the school and community and the power of school culture had the most influential impact on the effectiveness of the interventions (Love et al., 2019). Interviews from exemplar schools (i.e., those with relatively high active school travel rates) in New Zealand showed that there was an interactive relationship between school culture, community culture and the built environment, which were ‘common ingredients’ across these schools (Hawley et al., 2019). School representatives regarded the built environment around the school (e.g. walking and cycling infrastructure, traffic calming) as underpinning safety (i.e., traffic, personal) (Hawley et al., 2019, Smith et al., in press), the second strongest correlate of active school travel after distance to school (Ikeda et al., 2018a, Rothman et al., 2018). As Panter and Colleagues (2019) highlighted, qualitative or mixed method studies are often scarce and rated as low quality in systematic reviews on travel behaviour. However, these sources can provide insights into potential contexts and mechanisms of how interventions work, as well as in-depth examination of what factors influence a specific behaviour (Crawford and Garrard, 2013, Larouche et al., 2018, Panter et al., 2017, Smith et al., 2019a).

Little is known of how different school travel behaviours (e.g., walking, cycling/scootering/skateboarding, public transport, car) are associated with specific perceptions of and influences from environments (e.g., distance to school) (Mandic et al., 2017, Trapp et al., 2011). Public transport, for example, tends to be categorised as passive/motorised travel owning to its emissions and relative time spent being physically inactive (Ikeda et al., 2018b, Woodcock et al., 2009). However, it is argued that public transport is unique in terms of activity level (Rissel et al., 2012, Saelens et al., 2014, Villanueva et al., 2008, Voss et al., 2015) as well as environmental exposure and associated perceptions. Examining school travel behaviours separately may give novel and specific information of what facilitates and hinders each behaviour.

In this study, a multiphase mixed methods design was used to provide a comprehensive understanding of how school policies and practices supported and inhibited school travel behaviour in the context of Auckland, New Zealand. The study was guided by three objectives: (1) to identify school policies and practices related to school travel behaviour and traffic around school using interview data; (2) to contextualise emergent themes in relation to objectively measured child and school variables; and (3) to determine associations between school travel behaviour with objectively measured child and school variables.

## 2 Materials and methods

### 2.1 Study design and context

This study utilised a multiphase mixed methods design in which qualitative and quantitative components were implemented concurrently (e.g., data collection) as well as sequentially (e.g., data analysis). Data were drawn from Neighbourhoods for Active Kids, a cross-sectional study examining relationships between the built environment and a range of children’s activity behaviours and health outcomes in Auckland (most populated region in New Zealand with a third of the national population) (Statistics New Zealand, 2013). The full study design, methods and measures are described elsewhere (Oliver et al., 2016). Briefly, intermediate schools (junior high school, years 7–8, ages 10–13 years) in Auckland were selected based on a matrix of school decile (i.e., a measure of neighbourhood-level socioeconomic status), and child-specific measures of walkability (Giles-Corti et al., 2011) and destination accessibility (Badland et al., 2015) around each school’s geocoded address, with the purpose of increasing heterogeneity in these variables across the participating schools and their neighbourhoods. The diversity of geographic locations of the schools (north, south, east, west and central Auckland) was also taken into account. A neighbouring contributing primary school (elementary school, years 1–6, ages 5–11 years) for each intermediate school was also invited, to create a primary-intermediate school dyad in each neighbourhood.

A total of 19 state (i.e., government-funded and -operated) schools (nine intermediate and 10 primary) consented to participate in the study. Child data collection was conducted in the school setting, where the participants completed an online participatory geographic information systems (GIS) survey (https://maptionnaire.com) with one-on-one support from trained researchers (Kahila and Kyttä, 2009, Kyttä and Kahila, 2011, Kyttä et al., 2018). The survey included items on children’s usual mode of travel to school, their perceptions on neighbourhood and traffic safety, as well as mapping activity on their usual route to school. After the completion of child data collection, a computer-assisted telephone interview (CATI) survey was conducted with parents/caregivers of participating children. A face-to-face or telephone-based semi-structured interview with a school principal or representative from each school was conducted to explore school policies and practices related to school travel behaviour and traffic around school. Demographic information (i.e., school year (grade) and sex) of participating children were also reported by the school. All data from children, parents/caregivers and schools were collected between February 2015 and December 2016. On completion of the data collection, built environment attributes were objectively measured around the child’s school routes and school neighbourhood environment using GIS (e.g., Ikeda et al., 2018b, Ikeda et al., 2019). Ethical approval to conduct the study was granted by the host institution ethics committees (AUTEC, 14/263, 3 September 2014; MUHECN, 9 September 2014; UAHPEC, 9 September 2014).

### 2.2 Measures

Measures specific to the current study are described below.

#### 2.2.1 Child measures

Children reported their usual mode of travel to school using online participatory GIS by answering “*How do you usually get to school?*” with responses of ‘walk’, ‘bike’, ‘scooter (non-motorised)’, ‘skateboard’, ‘public transport’, or ‘car’. Due to the small number of bike (n = 42), scooter (n = 35) and skateboard (n = 6), these were combined into ‘wheel’ (n = 83), resulting in four categories in total: ‘walk’, ‘wheel’, ‘public transport’ and ‘car’. School year (5 to 8) and sex (male or female) were reported by schools, and ethnicity (classified as New Zealand European, Māori, Pacific, Asian, or other) by schools or parents/caregivers (if the school did not provide this information). Traffic safety perceptions were measured by two items (“*The roads around my school are busy with traffic before and after school*”; “*The roads around my school are full of parked cars before and after school*”) with a 4-point Likert scale (all of the time, most of the time, sometimes, hardly ever/never) (Mullan, 2003).

#### 2.2.2 School measures

##### 2.2.2.1 Socioeconomic status

A school decile is a relative rating of the socioeconomic position of a school community in New Zealand. Decile 1 indicates the 10% of schools with the highest proportion of students attending from areas of greatest socio-economic deprivation; while decile 10 schools are those with the lowest proportion of students attending from areas of greatest socio-economic deprivation (Ministry of Education, 2019). School decile ratings were categorised into three groups: low (deciles 1 to 3), medium (deciles 4 to 7) and high (deciles 8 to 10).

##### 2.2.2.2 Policy and practices

Nineteen semi-structured interviews were guided by two topics: (1) school policies and practices related to school travel behaviour, and (2) traffic around school. In relation to the first topic, a closed question about the involvement of active school travel programmes (e.g., Travelwise, walking school bus) was also asked (presence or absence).

Travelwise is a programme that has been developed through Safe School Travel Plans (i.e., an action plan for road safety and active travel) by Auckland Transport, and aims to create a safer traffic environment in the immediate school environment (Auckland Transport, 2019a). The Travelwise programme is supported by three pillars: curriculum (e.g., providing student-centred, curriculum based road safety, and active transport education programmes); ethos and organisation (e.g., reviewing policies, guidelines, and school traffic environment); and parents and community (e.g., providing parents with information and engaging school community and stakeholders) (Auckland Transport, 2019a). This needs-based approach could also involve infrastructural changes (e.g., installation of pedestrian crossings). The programme is delivered in collaboration with the school community, Auckland Council, New Zealand Police, New Zealand Transport Agency, and other organisations (Auckland Transport, 2019c). A walking school bus is a group of children walking to/from school under the minimum supervision of parent volunteers (Auckland Transport, 2019d). Walking school buses were not in operation for intermediate schools. The Travelwise programme was, however, in operation across primary, intermediate and secondary schools and, therefore, of particular interest in the current analysis.

##### 2.2.2.3 Built environment around the school

Objective measures of the built environment were generated in ArcGIS 10.2 (Environmental Systems Research Institute (ESRI), Redlands, California, USA) using an 800 m pedestrian network buffer area around each school address. The buffer scale of 800 m was selected based on the median distance (calculated using pedestrian network) of participating children who actively travelled to school (median: 800.3 m) (Ikeda et al., 2018b). The pedestrian network included all road classifications except motorways/state highways to best estimate possible environments for active travellers (Ikeda et al., 2018b).

Traffic exposure: Total lengths of high and low traffic roads (km) within the defined buffer were calculated. High (i.e., arterial rural/urban, major rural/urban and medium rural/urban) and low (i.e., low traffic roads included access rural/urban, minor rural/urban and foot path/track) traffic roads were determined based on road classification from the 2015 Corelogic Transport dataset as a proxy for traffic volume (Ikeda et al., 2019, Ikeda et al., 2018b).

School walkability: A school walkability index was generated using: (1) the ratio of the length of high to low traffic roads within the defined buffer (i.e., traffic exposure), and (2) the ratio of the pedestrian network area within the buffer area delineated using a Euclidian distance (of 800 m) to the Euclidian buffer area (i.e., Pedshed) (Giles-Corti et al., 2011). Both traffic exposure and Pedshed measures were collapsed into deciles, and the traffic exposure decile was reverse coded (i.e., 1 = most traffic to 10 = least traffic). The two measures were summed, resulting in scores ranging from 2 (least walkable) to 20 (most walkable) (Giles-Corti et al., 2011).

Child-specific neighbourhood destination accessibility index (NDAI-C): The NDAI-C is a weighted measure of accessibility based on the proportion of trips made to 28 neighbourhood destinations (e.g., schools, sports facilities, parks) by children (Badland et al., 2015). A binary scoring system was used to determine the presence or absence of each destination within the defined buffer. The NDAI-C (0 to 100) was calculated by the sum of values multiplied by the weight (post office: 0.04 to primary school: 50.65) and score (0 or 1) of each destination (Badland et al., 2015).

Cycle lane: Cycle lane length (CLL; km) derived from the Auckland Transport’s Open GIS Data Website (Auckland Transport, 2019b) and all road lengths (ARL; km) derived from the 2015 CoreLogic Transport dataset were generated within the defined buffer. The ratio of CLL/ARL was calculated, where a higher ratio denotes more cycle lanes available relative to all road types (Smith et al., 2019a).

Traffic lights: The ratio of traffic lights (i.e., controlled intersections) to area of the defined buffer was calculated (which were rescaled by multiplying by 10^6^). Traffic light data were obtained from the 2015 CoreLogic Transport dataset.

### 2.3 Data analysis

#### 2.3.1 Objective one

**Fig. 1:**
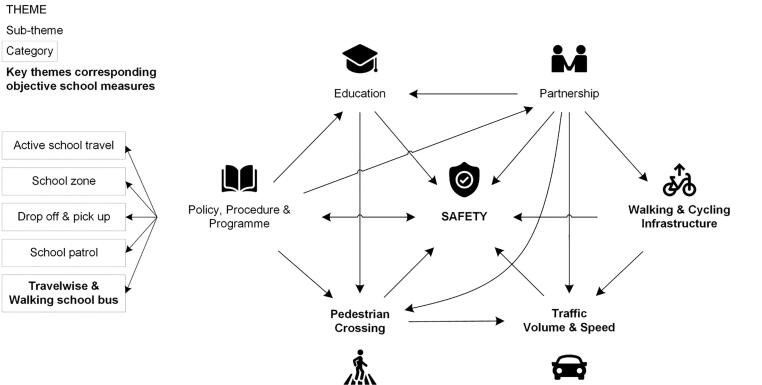
A thematic map of school policy and practices related to school travel behaviour and traffic around school.

Interviews with school principals and representatives were transcribed verbatim and thematic analysis was performed to find repeated patterns of meaning within the entire data (across individual interviews) (Braun and Clarke, 2006). Themes which captured important elements of school policy and practices related to school travel behaviour and traffic around school were identified inductively. The first author (EI) read and re-read transcripts to become familiarised with the data, developed an initial coding frame and coded data using NVivo (QSR International Pty Ltd. Version 12). After the refinement (e.g., adding, subtracting, combining) of the codes, all relevant codes were collated to form an overarching theme, sub-themes and categories, and to develop a thematic map (Fig. 1). The overarching theme, sub-themes and categories were reviewed by the last author (MS) to ascertain their accurate representation of the content of the entire data. In accordance with the objective of this mixed methods study, the themes were identified within the explicit meanings of the data (i.e., semantic content).

#### 2.3.2 Objective two

Out of all themes and categories identified in the qualitative data, the overarching theme, sub-themes and categories corresponding to objectively measured child and school variables were selected and used for further quantitative analyses to contextualise participants’ narrative accounts (Table 1) (e.g., Smith et al. (2019a)).

**Table 1:** An overarching theme, sub-themes, categories and their definitions and corresponding objectively measured child and school variables examined in this study.

Overarching theme					Corresponding objective measures	
*Name*		*Definition*			*Variable*	*Data source*
Safety		A state of being safe and not being in danger or at risk while travelling to/from school			Traffic safety perceptions	Child

**Sub-theme**			**Category**			
*Name*		*Definition*	*Name*	*Definition*		
Policy, procedure and programme		Policies, procedures and programmes that guide day-to-day operation of travelling to/from school	Active school travel	Rules for walking, cycling, scootering and/or skateboarding	–	–
			Drop off and pick up	Procedures for dropping off and picking up to/from school by cars	–	–
			School zone	Policy on an enrolment scheme (catchment area)	–	–
			School patrol	Procedure for controlling traffic flow and pedestrians at school crossing points	–	–
			Travelwise and walking school bus	Programmes for promoting active school travel	Travelwise programme	School
Traffic volume and speed		The volume and speed of vehicles observed around school	–	–	Traffic exposure	GIS
			–	–	School walkability	GIS
Pedestrian and cycling infrastructure		Infrastructure that provide safety and security for pedestrians and cyclists	–	–	Child-specific neighbourhood destination accessibility index	GIS
			–	–	Cycle lane	GIS
Pedestrian crossing		A special place in a road where vehicles must stop to allow people to walk/cycle across	–	–	Traffic lights	GIS
Education		The process of learning and training to acquire knowledge, skills, values, beliefs and habits	–	–	–	–
Partnership		The situation of individuals, communities, organisations and/or governments working together to create a safe environment for school travel behaviour	–	–	–	–

GIS = geographic information systems.

#### 2.3.3 Objective three

Mixed effects multinomial logistic regression models were used to account for the correlation structure of the data when determining associations between school travel behaviour and objectively measured child and school variables (Appendix A). The models were estimated using a quasi-maximum likelihood method, and Akaike Information Criterion (AIC) and Bayesian Information Criteria (BIC) were used as model fit indices. The outcome variable was school travel mode, in which car was a reference category and compared with walk, wheel, and public transport modes. The estimated intraclass correlation coefficients (ICCs) were calculated for neighbourhood and school to evaluate whether clustering by either neighbourhoods (primary-intermediate school dyad) or schools would account for a significant proportion of the total variance in school travel mode (i.e., the amount of correlation among individuals within the same neighbourhood or school) (Heck et al., 2012). The clustering effect was then accounted for by including a random intercept for that factor in all subsequent models. Model building was performed by first applying separate models for variables regarding children’s sociodemographics (Model 1a), children’s perceptions on traffic safety (Model 1b) and school measures (school decile, Travelwise and built environment around school: Models 2a-2c). All variables with a p-value < 0.1 from omnibus F tests were then added to the final model (Model 3). The base model (Model 1a) was kept constant in all models. To assess for potential multicollinearity between built environment attributes, variance inflation factor (VIF) and Pearson correlations were calculated. A *p*-value threshold of 0.05 was employed to determine statistical significance in the final model (Model 3). Sensitivity analyses were performed by comparing model fit in the final model with that in models with different random intercept and variable. All analyses were performed in SPSS (IBM Cooperation Version 25).

## 3 Results

### 3.1 Objective One: School policy and practices related to school travel behaviour and traffic around school

*Safety* was strongly embedded in school policy and practices and identified as an overarching theme (“The policy is, that obviously it’s vital the kids are safe first and foremost getting to and from school.”). This theme was related to six sub-themes and five categories (under the sub-theme of *policy, procedure and programme*), as outlined in Fig. 1 and Table 1. Safety concerns were evident in procedures around *active school travel* (e.g., students who cycle or scooter to school must wear a helmet and go through bike training) and *drop off and pick up* (“We have a drop off zone for parents with their cars and that’s, you know monitored.”). An issue of *school zone* was also discussed, where a large number of enrolments out of zone contributed to an increase in passive travel from/to school (“It’s the parents, the parents usually drop the children at school because most of our children arrive at school after receiving a lift because we have 85% of our roll live out of zone.”).

In terms of traffic safety, *pedestrian crossing* was identified as the place where most dangers and risks were involved partially due to high *traffic volume and speed* around school:

“A little kid standing with his scooter, he's waiting at the pedestrian crossing and the cars just keep zooming past him.”

Most schools set *school patrol* which are crossing points controlled and monitored by teachers and/or students:

“We have children working on the crossing so we encourage kids to cross there. It’s got a teacher manning it every day.”

The majority of schools organised road safety *education* for students which was primarily run in *partnership* with community police, Auckland Transport, and Auckland Council. A few schools addressed a need for educating parents:

“I guess our huge concern is sometimes the danger that parents expose the children to by the places that they cross. So, as I say we need the parent education.”

“We tell them [parents] we don't want them to be dropping them [children] off in the bus bay, we have the pedestrian crossing for the children to use in the morning to get from the other side to here, and we would prefer that they, the parents who are dropping the children off, did not use the bus bay where buses are coming in, dropping children off.”

School awareness of the *Travelwise and walking school bus* were recounted by all school principals and representatives. Compared to the Travelwise programme (which works with primary, intermediate and secondary schools, and is run by Auckland Transport and other collaborators), walking school buses were predominantly operated in primary schools, and heavily rely on parental support.

“We do have a walking school bus or two that run and they’re [run] really well, those parents that run those are really committed to that work.”

Advantages of the Travelwise programme were highlighted in terms of the provision of *pedestrian crossings* and *walking and cycling infrastructure* around school (e.g., cycle lanes, traffic calming and islands) and the encouragement of active school travel. The patterns of *pedestrian crossing* were differentiated from *walking and cycling infrastructure* because school principals and representatives exclusively and explicitly delivered a narrative of pedestrian crossings particularly in relation to *policy, procedure and programme* and *education* (Fig. 1).

“Well,…there’s been things done with the Travelwise and they’ve now been fiddling around here with the pedestrian crossing. They’re just putting in the crossing that we’ve been trying to get for 20 years.”

“The Travelwise team get out there and have special days where the kids walk, a walking day or they’ll have a day where if you walk you get a sticker at the gate or there’s people waiting for you at the gate with balloons or something if you walk to school you get something, so that all encourages them.”

School principals and representatives believed that a dearth of pedestrian crossings and cycle lanes around school was a critical barrier to active school travel.

### 3.2 Objective two: Contextualisation of the overarching theme, sub-themes and categories

The overarching theme, sub-themes and categories identified from the qualitative data reflected similarities in issues of, and strategies for, school travel behaviour across schools. Despite these commonalities, differences in school travel behaviour between schools existed (Appendix B), indicating the provision of quantifiable data to contextualise these overarching theme, sub-themes and categories was warranted. The overarching theme, sub-themes and categories were selected based on the availability of objectively measured child and school variables in the Neighbourhoods for Active Kids study (Table 1), which informed quantitative modelling.

### 3.3 Objective three: Associations between school travel behaviour and objectively measured child and school variables

#### 3.3.1 Characteristics of participating children and schools

Data from 1085 children across 19 schools were included in analyses. Descriptive information for objectively measured child and school variables is presented in Table 2. Just under half (46.1%) of children were driven to school, and 34.3% walked. A minority (7.6%) used wheels with half of these children cycling to school (3.9%). Over half of children in years 5 (58.3%) and 6 (53.8%) travelled by car compared with approximately one-third of those in years 7 (35.0%) and 8 (36.7%). Boys were more likely to actively travel to school (walk: 37.8%, wheel: 12.2%) than girls (walk: 31.1%, wheel: 3.3%). With the exception of New Zealand European children (whose most common mode was walking), car travel was the most common travel mode to school across the ethnic groups.

**Table 2:** Descriptive information of objectively measured child and school variables.

Variable	Description	Measurement scale	Descriptive statistics^†^
*Child measures (N = 1085)*			
School travel mode	How do you usually get to school	Walk	34.4%
		Wheel	7.6%
		Public transport	11.9%
		Car	46.1%
Year	Child's school year	Year 5	24.5%
		Year 6	26.4%
		Year 7	24.2%
		Year 8	24.9%
Sex	Child's sex	Male	49.0%
		Female	51.0%
Ethnicity	Child's ethnicity	NZ European	40.9%
		Māori	12.7%
		Pacific people	15.2%
		Asian	13.5%
		Other	17.7%
Traffic safety^‡^	1. The roads around my school are busy with traffic before and after school	All of the time	13.0%
		Most of the time	40.7%
		Sometimes	37.2%
		Hardly ever/Never	8.8%
	2. The roads around my school are full of parked cars before and after school	All of the time	17.1%
		Most of the time	36.2%
		Sometimes	35.9%
		Hardly ever/Never	10.3%
*School measures (N = 19)*			
School decile	Neighbourhood-level socioeconomic position	Low	29.1%
		Medium	22.9%
		High	48.0%
Travelwise	The presence of the Travelwise programme	Yes	75.2%
		No	24.8%
High traffic exposure	Length of high traffic roads (km) within a 800 m buffer around school	–	8.2 ± 3.8
Low traffic exposure	Length of low traffic roads (km) within a 800 m buffer around school	–	18.7 ± 5.7
School walkability	A composite index of Pedshed* and ratio of high to low traffic exposure within a 800 m buffer around school	2–20	11.0 ± 3.9
NDAI-C	A weighted index of accessibility to neighbourhood destinations for children within a 800 m buffer around school	0–100	59.2 ± 21.2
Cycle lane	Ratio of cycle lane length to all roads length within a 800 m buffer around school	–	0.2 ± 0.1
Traffic lights	Ratio of number of traffic lights (controlled intersections) to the land area of a 800 m buffer around school (/10^6^)	–	0.8 ± 1.3

NDAI-C = child-specific neighbourhood destination accessibility index.

†Frequencies (%) for binary or ordinal variables; mean ± standard deviation for continuous variables.

‡Missing data (busy traffic: n = 2; parked cars: n = 4) were excluded.

*A ratio of the pedestrian network area within the buffer area delineated using a Euclidian distance (of 800 m) to the Euclidian buffer area. A higher ratio indicates a more connected streets for pedestrians.

#### 3.3.2 Model building using mixed effects multinomial logistic regression models

**Fig. 2:**
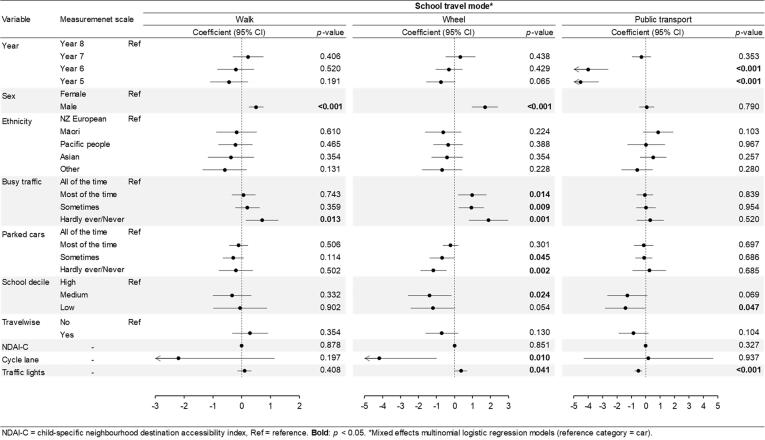
Final model of associations between school travel behaviour and objectively measured child and school variables using mixed effects multinomial logistic regression models (N = 1081).

The overall procedure of model building is presented in Appendix A. The assessment of ICC showed little evidence of variability in the school travel mode between neighbourhoods but a significant proportion of variance between schools. A random intercept for school was, therefore, included in all mixed effects multinomial logistic regression models. The estimated proportions of variance in school travel mode between schools were 13.92% (95% confidence interval (CI): 6.77–26.50%) for walk versus car, 27.83% (95% CI: 13.68–48.40%) for wheel versus car, and 50.10% (95% CI: 30.54–69.63%) for public transport versus car. Potential multicollinearity was detected from VIF and Pearson correlations which resulted in removing lengths of low traffic roads from the built environment measures. A listwise deletion method was used to handle missing data in child traffic safety variables (busy traffic: n = 2, parked cars: n = 4 including two cases also missing busy traffic data), resulting in the total of 1081 cases in Models 1b (child traffic safety) and 3 (final model). Model convergence was achieved for all models. In the model building process, all objectively measured child and school variables except length of high traffic roads (*p* = 0.533) and school walkability (*p* = 0.129) reached the significance level at *p* < 0.1 in omnibus F tests (Appendix C). Ten variables were therefore included in the final model where results were fully adjusted (Fig. 2 and Appendix D as a table format). The robustness of the estimates in the final model was demonstrated through sensitivity analyses where model fit (i.e., AIC, BIC) in the final model was better than that in models with a neighbourhood random intercept or all variables (including high traffic exposure and school walkability) (Appendix E). There was evidence of variation in the random intercepts, implying differences in patters of school travel mode between schools. The variance between schools was similar for active travel compared to car (estimated variance of school intercepts: 0.69, 95% CI: 0.25–1.90 for walk versus car; 0.67, 95% CI: 0.16–2.87 for wheel versus car), but a greater estimated variance was observed for public transport compared to car (2.19, 95% CI: 0.66–7.22). Children who actively travelled to school were less likely to report busy traffic around schools compared with those driven to school (walk: *p* = 0.013, wheel: *p* = 0.001) in which the probability of cycling/scootering/skateboarding (compared to being driven) had a negative association with the perception of busy traffic around school. Awareness of parked cars was lower among children driven to school than those using active travel modes. Students from mid-decile school communities were less likely to cycle/scooter/skateboard to school than those in high-decile neighbourhoods (*p* = 0.024). Students from schools in most deprived neighbourhoods had lower odds (versus those in high-decile schools) of using public transport rather than cars (*p* = 0.047). The ratio of cycle lane to all road length was negatively associated with cycling/scootering/skateboarding to school. Students from the schools with a higher ratio of number of traffic lights around were more likely to use bike/scooter/skateboard to school; whereas those from the schools with a lower rate of traffic lights tended to travel more by public transport than by cars.

## 4 Discussion

The overall aim of this research was to provide a comprehensive understanding of how school policies and practices supported or inhibited school travel behaviour in the context of Auckland, New Zealand. Using multiphase mixed methods, three approaches were undertaken to achieve this aim. Firstly, an overarching theme, sub-themes and categories were generated from school principal/representative interviews about school travel policies, practices and traffic around school. Secondly, the overarching theme, sub-themes and categories were contextualised using corresponding objectively-measured child and school variables to develop quantitative modelling. Thirdly, quantitative modelling was undertaken to identify associations between school travel behaviour and objectively measured child and school variables.

Thematic analysis revealed that all schools had specific policies, procedures and programmes of school travel behaviour that were primarily designed to ensure student’s safety. Resources such as the Travelwise programme, pedestrian crossings to control traffic around school and road safety education were collaboratively supplied by Auckland Council, Auckland Transport, New Zealand Police and other organisations. Consistent with these qualitative findings, results from mixed effects multinomial logistic regression models indicated significant associations between school travel behaviour and the emergent overarching theme and sub-themes of *safety*, *walking and cycling infrastructure* and *pedestrian crossing*. Those cycling, scootering or skateboarding to school were more aware of parked cars. Active travellers were likely to perceive their immediate school environment to have lower levels of busy traffic than those chauffeured to school. The probability of cycling, scootering and skateboarding (versus being driven) was negatively associated with a ratio of length of cycle lanes to all roads, and positively associated with a higher ratio of traffic lights.

The quantitative modelling also shed light on differences in school travel behaviour by child and school sociodemographic characteristics. Compared to intermediate school-aged children, primary school-aged children were less likely to use public transport than car. The odds of active travelling to school were higher in boys than girls. The results from this study were consistent with nationally representative data where the prevalence of active travel was higher in older children and boys than the counterparts (46.3% (boys) versus 42.6% (girls) in the New Zealand Health Survey; 36% (ages 5–7) versus 49% (ages 12–14) in the Active New Zealand Youth Survey) (Smith et al., 2019b). There was a higher likelihood of cycling, scootering, skateboarding and public transport use than car travel in children attending high-decile schools.

### 4.1 Associates of how children perceived traffic safety

Interestingly, children who actively travel to school were less likely to perceive busy traffic but more likely to report parked cars around schools than those driven to school, as indicated in the quantitative data. Coupling with the overarching *safety* theme, these findings imply perceptions of traffic safety in relation to school travel mode may be behaviour- and context-specific. For example, children who were driven to school might have more sensitivity to motorised traffic conditions because they experienced more traffic congestion than those who actively travelled to school (Egli et al., 2019). In active travellers, traffic danger might be experienced mostly at pedestrian crossings and around drop-off/pick-up areas where a concentration of cars roamed and parked, as identified in the qualitative data. In this respect, school patrols might have played a vital role in improving traffic safety at pedestrian crossings, which subsequently lowered the level of busy traffic perceptions around school. Moreover, traffic safety perceptions are influenced by not only the volume and speed of traffic but also other elements such as public surveillance of the setting (Egli et al., 2018). The current quantitative modelling did not show an association between GIS-derived objective measures of *traffic volume and speed* within the immediate school environment (i.e., length of high and low traffic roads within an 800 m buffer around school) and school travel behaviour. These measures were based on the road classification which might not reflect the actual flow of traffic (e.g., traffic congestion) during the morning rush hour. This limitation demonstrates the importance of capturing both objective and subjective perspectives of safety of traffic environments. For instance, children living in San Diego, USA perceived busy commercial areas with more traffic as safer walking environments than quiet and remote residential roads with less traffic (Jamme et al., 2018, McGrath et al., 2016). Further qualitative research to investigate what and which settings are perceived as facilitators or barriers to traffic safety with which travel mode may be required to develop quantitative data. Information about behaviour- and context-specific traffic safety can contribute to the development of measurement which can specifically and sensitively assess traffic safety for school travel behaviour.

### 4.2 Built environments acting as facilitators or barriers to school travel behaviour

An inverse relationship between objectively measured cycle lanes and active school travel behaviour appears counter-intuitive at first. However, this finding was somewhat expected based on previous examinations of cycle lane availability and quality in relation to parent neighbourhood environment perspectives in Auckland (Smith et al., 2019a). These findings suggest that cycle lanes, compared to cycle paths (i.e., segregated tracks/trails for cyclists and/or pedestrians), in the Auckland context might not be designated for children to safely cycle, scooter or skateboard on. In fact, it has been suggested that cycle lanes are suitable for ‘enthused and confident’ cyclists (NZ Transport Agency, 2019), rather than young, school-aged children. Cycle lanes are, however, more commonly constructed than cycle paths in Auckland (Auckland Transport, 2019b). According to New Zealand law, bicycles with wheels larger than 355 mm diameter (e.g., a tricycle or small child’s bicycle) are not allowed to be ridden on footpaths, whereas scooters and skateboards are legally allowed on the footpath regardless of their wheel size (NZ Transport Agency, 2017). The New Zealand Police recommend that children under the age of 10 years should not cycle on the road without supervision despite no legality regarding the minimum age of cycling on the road (New Zealand Police, 2019). Correspondingly, the New Zealand Transport Agency advises that being over the age of 11 years may be appropriate for children to cycle on the road unsupervised (NZ Transport Agency, 2013). Under these circumstances, children and parents need to make the ultimate decision about how to cycle to school or other destinations: either breaking the law by cycling on the footpath, or taking the risk of using the cycle lane adjacent to high volume vehicles (particularly during the morning rush hour) (Randal et al., 2018). Children, especially younger children may often use a safe footpath rather than an unsafe ‘painted strip on a road’. Given the impact of built environment changes (e.g., new infrastructure for walking and cycling) on people’s perceptions and behaviour (Panter and Ogilvie, 2015, Prins et al., 2016), investment and improvement in dedicated cycling infrastructure (e.g., Cambridge Cycling Campaign (2014)) may be a potential solution to this problem.

**Fig. 3:**
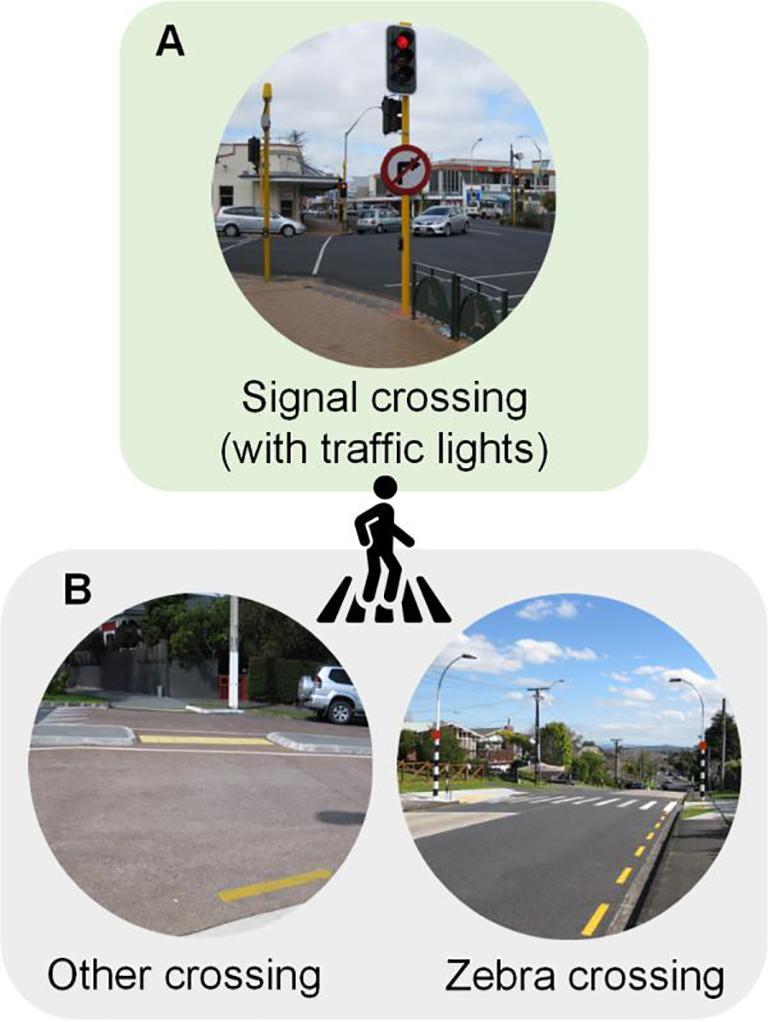
Different types of pedestrian crossings. A = Crossings included in the data source in GIS. B = Crossings excluded from the data source in GIS.

The ratio of number of traffic lights (controlled intersections) to the immediate area around school was positively associated with higher odds of cycling/scootering/skateboarding than being driven, indicating a need for signalised crossings (i.e., crossings controlled by traffic lights) for children to travel to school by wheeled modes. In the interviews with school principals and representatives, however, pedestrian crossings did not always refer to signal crossings but zebra crossings and other crossings (without traffic lights; where school patrols were arranged) (Fig. 3). Larouche and colleagues (2014a) reported the presence of crossing guards (i.e., school patrols) was positively associated with active school travel. In New Zealand, GIS data sources for the zebra and other crossings were not available at the time of this study. Therefore, in light of data availability, the *pedestrian crossing* sub-theme was limited to signal crossings (at traffic lights) and school staff accounts. Latest remote sensing techniques may be a potential method to capture different types of pedestrian crossings using aerial imagery (Hua et al., 2019). These advances in measurement of traffic safety and built environments can benefit the evaluation of interventions such as Travelwise programmes.

### 4.3 Safe environments for children’s active school travel

The Safe School Travel Plans (part of the wider Travelwise programme) integrate multiple strategies of engineering, education, enforcement, encouragement and policy into an action plan to promote active school travel (Hinckson and Badland, 2011, Hinckson et al., 2011). This multidisciplinary scheme was acknowledged by participating school principals and representatives under the sub-themes of *policy, procedure and programme*, *pedestrian crossing*, *walking and cycling infrastructure* and *education* (Fig. 1). The Canadian site of the International Study of Childhood Obesity, Lifestyle and the Environment (ISOCOLE) also showed an interaction effect for Safe Routes to School programme and traffic calming (i.e., speed bumps, narrower lanes) to facilitate active school travel (Larouche et al., 2014a). A pivotal role of community settings beyond the immediate school environment was highlighted in which positive community culture, neighbourhood social interactions and relationships between school and community can reinforce active school travel (Hawley et al., 2019, Smith et al., 2020). Auckland Transport recently put forward their Safer Communities programme in the Auckland Regional Land Transport Plan 2018–2028 wherein consolidated (e.g., creating and improving streets and footpaths for active travellers, supporting safer driving behaviours) and community-based approaches were encompassed (Auckland Transport, 2018a, Transport, 2018b). Aligned with the C-STBM (Ikeda et al., 2019) and the World Health Organization’s guiding principles of creating active societies, environments (World Health Organization, 2018), people and systems through all levels from upstream (i.e., social and environmental factors) to downstream (i.e., individual factors), this multifaceted approach may be key to creating safe environments and promoting active school travel.

### 4.4 Need for equitable opportunities of school travel behaviour

Our findings drew some attention to the issue of inequity (i.e., unfair disparities derived from social, cultural, economic and environmental conditions that can be improved upon by human action) (World Health Organization, 2018). Significantly lower odds of wheeling or public transport in children from low-to-mid-decile schools may indicate that children living in communities of higher deprivation might not have sufficient access to these modes of travel. Along with school attitudes towards discouraging students to cycle to school, the accessibility and affordability of bike, scooter, skateboard and public transportation need to be taken into account to promote such travel modes. The Bike On New Zealand Charitable Trust implemented the Bikes in Schools programme in which primary schools purchase a package of bikes, helmets, tracks, bike storage and cycle skills training cost-effectively to promote cycling in children (Bike On New Zealand Charitable Trust, 2019). Schools are liable to secure funders to cover the cost of equipment (e.g., approximately NZ$60,000 for an average school of 300 students) and ongoing maintenance (approximately NZ$3,000) (Bike On New Zealand Charitable Trust, 2019). The programme, however, is not specifically designed to support low-to-mid-decile schools.

Auckland Transport has delivered the Bike Safe training for years 5 and 6 students (ages 9–11 years) in primary schools since 2010 to improve their cycle skills. Although the training can provide equal opportunities for students across high-to-low decile schools to improve their skills as well as self-efficacy of cycling, the issue of inequities of access to a bike (or scooter or skateboard) has still remained. Recently Auckland Council proposed a new NZ$1.1 million initiative to support public transport wherein free public transport (including buses, trains and ferries) has been available on weekends for all children under 16 years since September 2019 (Wilson, 2019). This initiative also potentially provides equal opportunities for children to build up their experience, skills and confidence of using the service, which can subsequently lead to a rise in the use of public transport in this population (Goodman et al., 2014). Notwithstanding, an issue still exists in terms of the affordability of using public transport to travel to/from school on weekdays particularly for those from low socioeconomic status families. The current study did not differentiate school buses (fare/no fare) from public buses (fare). Further research may be required to investigate the influence of different types of public transport services (e.g., school bus versus public bus) on school travel behaviour. The supply of subsidised school buses targeting at low-to-mid-decile schools may reduce inequities of access to public transport particularly for bus users (Wilson et al., 2010).

The prevalence of the Travelwise programme in the schools in the current study (75.2%) was equivalent to programme coverage for all Auckland school-aged children (75%) (Auckland Transport, 2019a). However, the level of engagement in the programme varied by school: from a lower level of commitment (“We have been in the Travelwise group but really there’s not a lot to do here.”) to a higher level of commitment (“We have a gold [award]” (i.e., ‘gold’ is the highest award of the programme given to schools that made significant contribution to reduction in car travel and traffic congestion as well as improvement in road safety)). To fill this gap, Auckland Transport shifted their focus and efforts from recruiting all schools (quantity-focus) to developing a comprehensive approach to support school communities with greatest need (quality-focus), which may mitigate the inequity of active school travel (Auckland Transport, 2015). This approach, particularly in the process of selecting school communities, may need to cautiously consider the balance of social and environmental needs – for instance, a focus of reducing environmental inequities (e.g., in rural schools) may exacerbate social inequities further (e.g., rural communities with high socioeconomic status).

### 4.5 Understanding of the role of school policies and practices in school travel behaviour

**Fig. 4:**
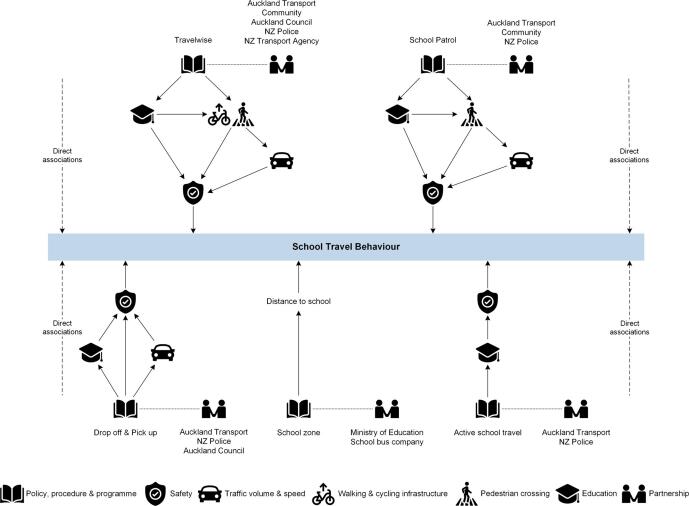
Example associations of school policy and practices related to school travel behaviour. NZ = New Zealand. Each framework outlines relationships between the category of *school policy, procedure and programme* and the other sub-themes. Potential direct associations between each sub-theme/category and school travel behaviour were indicated in dotted lines.

This study addressed the important role of school policies and practices that may facilitate or hinder children’s school travel behaviour. For example, robust Travelwise programmes, school patrol procedure, and active school travel rules may facilitate children’s active school travel; however, lenient school zone policy (e.g., allowing children living outside the school zone to enrol at the school) and drop-off and pick-up procedure (e.g., allowing parents to drive in the school site) may hinder this behaviour. In fact, school enrolment schemes in New Zealand tend to act as ‘guidelines’, and parents are likely to choose their children’s schools based on school quality and reputation rather than proximity (Ministry of Education, 2017, Morton et al., 2018). Fig. 4 outlines specific relationships between the sub-themes of *school policy, procedure and programme* and the overarching and other sub-themes based on the thematic map (Fig. 1). Each sub-theme of the school policies, procedures and programmes were distinctively, but to some extent similarly, interrelated with the overarching and other sub-themes. One of the fundamental ingredients of these example associations was a multidisciplinary team who can provide school support for active school travel. Each association may work independently, but interactive and collective benefits may be produced by applying more than one association.

Children’s school travel behaviour may be associated with the environment beyond the school settings (e.g., route environment (Ikeda et al., 2018b)). As Ikeda and colleagues (2019) elucidated, the role of school environment is one of seven domains that had direct and indirect associations with school travel behaviour. Given the integral role of the other six domains (i.e., built environment, social environment, household characteristics, household beliefs, child characteristics, child beliefs) in school travel behaviour, a holistic approach including environmental (school, social, built), household and child factors may be needed to support changes in school travel behaviour from passive to active travel.

## 5 Strengths and limitations

This study employed multiphase mixed methods to provide a comprehensive understanding of how school policies and practices supported and inhibited school travel behaviour. To our knowledge, this is the first study to integrate findings from (1) interviews with school principals or representatives about school policy and practices related to school travel behaviour and (2) mixed effects multinomial logistic regression models using objectively measured child and school variables. The mixed methods allowed us to triangulate qualitative and quantitative findings, which led to in-depth interpretation of the overall findings.

Despite these strengths, this study was cross-sectional and conducted in only the urbanised Auckland region in New Zealand; therefore, findings cannot be generalised to other school environments and causality cannot be inferred. Due to a small sample size of the wheel group (7.6%), particular comparisons with this group may lack statistical power. GIS-derived built environment measures were calculated within the immediate school environment defined as 800 m pedestrian network buffer around school. To minimise exposure misclassification (Pannucci and Wilkins, 2010), the buffer size was cautiously (rather than arbitrarily) determined based on the median distance of participating children who actively travelled to school (Ikeda et al., 2018b) and previous studies (around school (e.g., Braza et al. (2004)), around home (e.g., Panter et al. (2010)). Furthermore, school entrance locations (points) were manually identified by the first author (EI) during the data collection, and the school site (polygon) was delineated using the points of access to the school (rather than single centroid) (Harrison et al., 2014, Ikeda et al., 2018b). The size/area of school zones (nzschools.tki.org.nz) were also visually inspected. No patterns were observed in terms of school decile but school type (where intermediate schools tended to have bigger school zones than primary schools). Nevertheless, the issues of spatial scale and zoning (i.e., modifiable area unit problem) as well as spatial and temporal uncertainty relating to actual exposure (i.e., uncertain geographic context problem) mean that findings should be interpreted with caution.

## 6 Conclusions

Road traffic safety for children was the primary goal of school policy and practices related to school travel behaviour in Auckland, New Zealand. To facilitate active school travel, it is essential for schools to deliver road safety education and skills training, to have secure crossings and other walking and cycling infrastructure, and for traffic to be controlled around the school. The implementation of school policies, procedures and programmes including the Travelwise programme was predominantly supported by national and regional governments, local councils and school communities. Nevertheless, further support from these partners may be required to provide equitable opportunities to travel to school by bike and public transport (including school buses) for children with low-to-mid socioeconomic status in New Zealand. Future research is needed to develop behaviour- and context-specific measures of traffic safety perception and the built environment around school for use in school travel behaviour. Overall, this study has demonstrated the important role of school policy and procedures in relation to school travel behaviour and provided recommendations for future interventions to use an intersectoral approach to support changes in school travel behaviour.

## CRediT authorship contribution statement

**Erika Ikeda:** Conceptualization, Methodology, Formal analysis, Investigation, Writing - original draft, Writing - review & editing, Visualization. **Suzanne Mavoa:** Conceptualization, Data curation, Writing - review & editing, Funding acquisition. **Alana Cavadino:** Conceptualization, Methodology, Formal analysis, Writing - review & editing. **Penelope Carroll:** Conceptualization, Methodology, Formal analysis, Investigation, Writing - review & editing, Funding acquisition. **Erica Hinckson:** Conceptualization, Methodology, Writing - review & editing, Supervision. **Karen Witten:** Conceptualization, Methodology, Writing - review & editing, Supervision, Funding acquisition. **Melody Smith:** Conceptualization, Methodology, Formal analysis, Writing - review & editing, Supervision, Project administration, Funding acquisition.
