# Supplementary material for: Keeping kids safe for active travel to school: A mixed method examination of school policies and practices and children’s school travel behaviour
Source: Travel Behav Soc. 2020 Oct;21:57–68. doi: 10.1016/j.tbs.2020.05.008 (PMC7473447; doi:10.1016/j.tbs.2020.05.008)
Supplement: Supplementary data 1 [file mmc1.docx]

# Supplementary files

Appendix A: The procedure of model building using the generalised linear mixed models with multinomial logistic regression


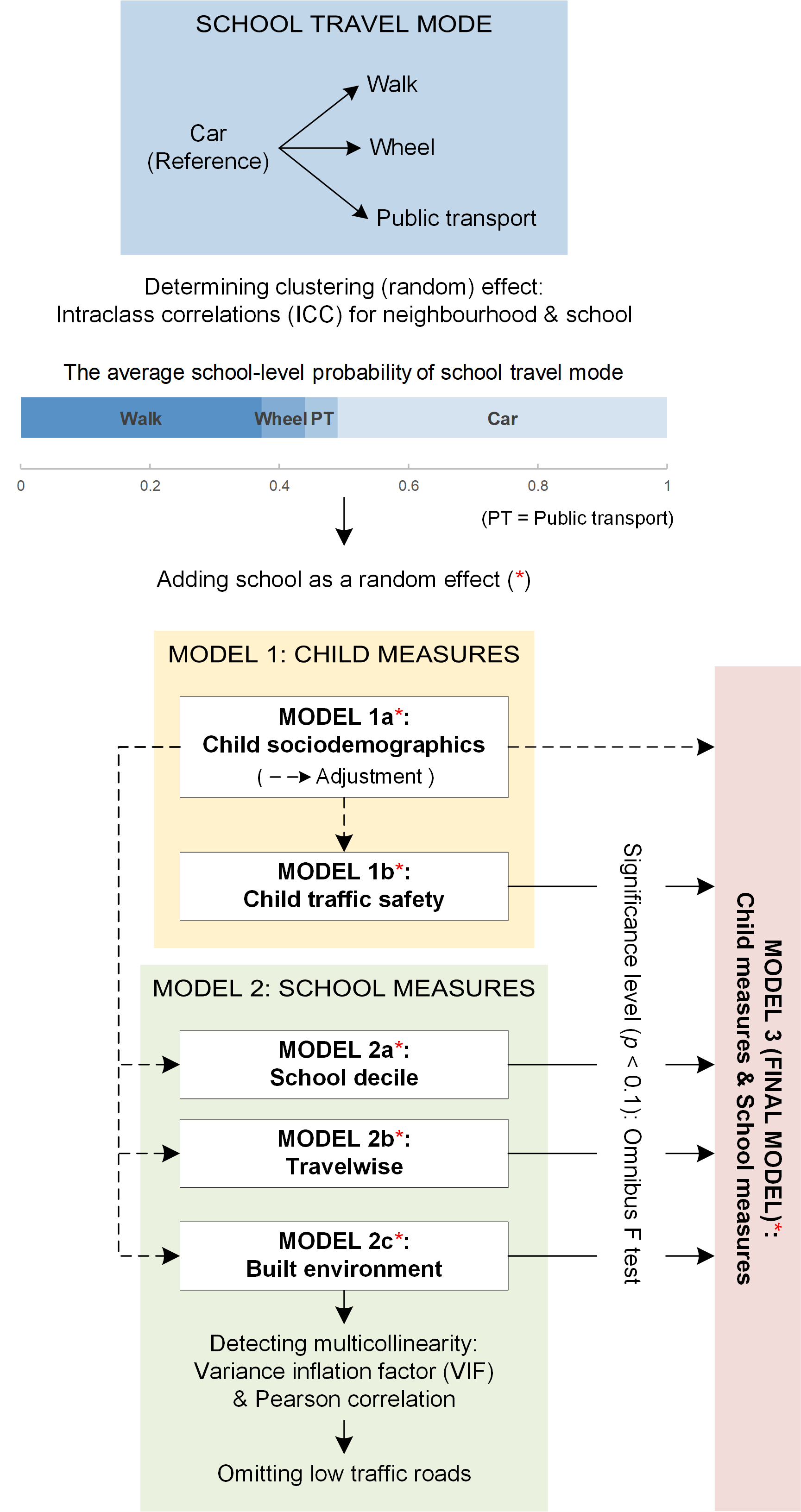


Appendix B: The distribution of school travel mode by school

| School* | School type | Participant  (N) | School travel mode (%) | | | | | |
| --- | --- | --- | --- | --- | --- | --- | --- | --- |
|  |  |  | 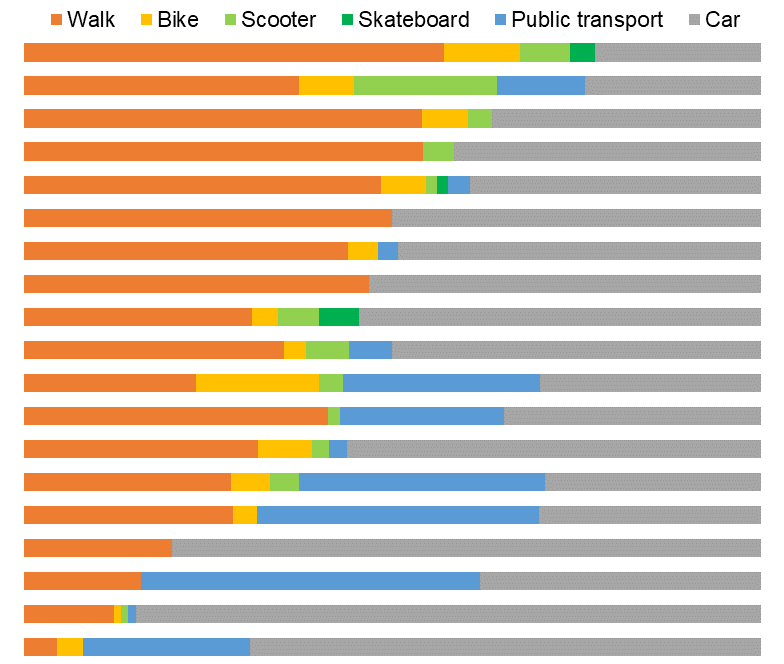Walk | Bike | Scooter | Skateboard | Public transport | Car |
| 1^a^ | Primary | 58 | 56.9 | 10.3 | 6.9 | 3.4 | 0.0 | 22.4 |
| 2^a^ | Intermediate | 67 | 37.3 | 7.5 | 19.4 | 0.0 | 11.9 | 23.9 |
| 3^b^ | Primary | 63 | 54.0 | 6.3 | 3.2 | 0.0 | 0.0 | 36.5 |
| 4^c^ | Primary | 72 | 54.2 | 0.0 | 4.2 | 0.0 | 0.0 | 41.7 |
| 5^d^ | Intermediate | 66 | 48.5 | 6.1 | 1.5 | 1.5 | 3.0 | 39.4 |
| 6^d^ | Primary | 24 | 50.0 | 0.0 | 0.0 | 0.0 | 0.0 | 50.0 |
| 7^e^ | Intermediate | 75 | 44.0 | 4.0 | 0.0 | 0.0 | 2.7 | 49.3 |
| 8^e^ | Primary | 47 | 46.8 | 0.0 | 0.0 | 0.0 | 0.0 | 53.2 |
| 9^f^ | Primary | 55 | 30.9 | 3.6 | 5.5 | 5.5 | 0.0 | 54.5 |
| 10^g^ | Intermediate | 34 | 35.3 | 2.9 | 5.9 | 0.0 | 5.9 | 50.0 |
| 11^f^ | Intermediate | 30 | 23.3 | 16.7 | 3.3 | 0.0 | 26.7 | 30.0 |
| 12^h^ | Intermediate | 63 | 41.3 | 0.0 | 1.6 | 0.0 | 22.2 | 34.9 |
| 13^i^ | Primary | 41 | 31.7 | 7.3 | 2.4 | 0.0 | 2.4 | 56.1 |
| 14^b^ | Intermediate | 75 | 28.0 | 5.3 | 4.0 | 0.0 | 33.3 | 29.3 |
| 15^c^ | Intermediate | 60 | 28.3 | 3.3 | 0.0 | 0.0 | 38.3 | 30.0 |
| 16^d^ | Primary | 20 | 20.0 | 0.0 | 0.0 | 0.0 | 0.0 | 80.0 |
| 17^i^ | Intermediate | 63 | 15.9 | 0.0 | 0.0 | 0.0 | 46.0 | 38.1 |
| 18^g^ | Primary | 99 | 12.1 | 1.0 | 1.0 | 0.0 | 1.0 | 84.8 |
| 19^h^ | Primary | 88 | 4.5 | 3.4 | 0.0 | 0.0 | 22.7 | 69.3 |
| N = number. *A neighbourhood (primary-intermediate school dyad) is shown as a superscripted lowercase letter.  Note. Schools are listed in order of high to low prevalence of active school travel (i.e., walk, bike, scooter, skateboard). | | | | | | | | |

Appendix C: Results of omnibus F tests in the model building process

|  | Variable | F^†^ | df1 | df2 | *p*-value |
| --- | --- | --- | --- | --- | --- |
| Model 1a: Child sociodemographics | | | |  |  |
|  | Year | 13.26 | 9 | 1058 | **<0.001** |
|  | Sex | 9.92 | 3 | 1058 | **<0.001** |
|  | Ethnicity | 2.30 | 12 | 1058 | **0.007** |
| Model 1b*: Child traffic safety | | |  |  |  |
|  | Busy traffic | 1.78 | 9 | 1036 | **0.069** |
|  | Parked cars | 2.21 | 9 | 1036 | **0.020** |
| Model 2a*: School decile | |  |  |  |  |
|  | School decile | 8.70 | 6 | 1052 | **<0.001** |
| Model 2b*: Travelwise | |  |  |  |  |
|  | Travelwise | 7.79 | 3 | 1055 | **<0.001** |
| Model 2c*: Built environment^‡^ | | |  |  |  |
|  | High traffic exposure | 0.73 | 3 | 1043 | 0.533 |
|  | School walkability | 1.89 | 3 | 1043 | 0.129 |
|  | NDAI-C | 3.91 | 3 | 1043 | **0.009** |
|  | Cycle lane | 6.73 | 3 | 1043 | **<0.001** |
|  | Traffic lights | 7.05 | 3 | 1043 | **<0.001** |
| NDAI-C = child-specific neighbourhood destination accessibility index. **Bold**: *p* < 0.1 | | | | | |
| ^†^A random intercept for school was added to account for school clustering. | | | | | |
| *Models were adjusted for child sociodemographic characteristics (year, sex and ethnicity) | | | | | |
| ^‡^Low traffic exposure was omitted due to multicollinearity. | | | | | |

Appendix D: Final model of associations between school travel behaviour and objectively measured child and school variables using mixed effects multinomial logistic regression models (N = 1081)

| Variable | Measurement scale | **Walk** | | **Wheel** | | **Public transport** | |
| --- | --- | --- | --- | --- | --- | --- | --- |
|  |  | Coefficient* (95% CI) | *p*-value | Coefficient* (95% CI) | *p*-value | Coefficient* (95% CI) | *p*-value |
| Year | Year 8 | Reference |  | Reference |  | Reference |  |
|  | Year 7 | 0.22 (-0.31, 0.76) | 0.406 | 0.33 (-0.50, 1.16) | 0.438 | -0.31 (-0.96, 0.34) | 0.353 |
|  | Year 6 | -0.21 (-0.84, 0.42) | 0.520 | -0.30 (-1.05, 0.45) | 0.429 | -4.01 (-5.41, -2.60) | **<0.001** |
|  | Year 5 | -0.44 (-1.09, 0.22) | 0.191 | -0.77 (-1.58, 0.05) | 0.065 | -4.52 (-5.76, -3.28) | **<0.001** |
| Sex | Female | Reference |  | Reference |  | Reference |  |
|  | Male | 0.50 (0.25, 0.75) | **<0.001** | 1.69 (0.97, 2.41) | **<0.001** | 0.07 (-0.45, 0.59) | 0.790 |
| Ethnicity | NZ European | Reference |  | Reference |  | Reference |  |
|  | Māori | -0.18 (-0.88, 0.51) | 0.610 | -0.63 (-1.64, 0.39) | 0.224 | 0.87 (-0.18, 1.91) | 0.103 |
|  | Pacific people | -0.22 (-0.80, 0.37) | 0.465 | -0.36 (-1.17, 0.46) | 0.388 | 0.03 (-1.27, 1.32) | 0.967 |
|  | Asian | -0.38 (-1.18, 0.42) | 0.354 | -0.41 (-1.28, 0.46) | 0.354 | 0.52 (-0.38, 1.43) | 0.257 |
|  | Other | -0.59 (-1.35, 0.18) | 0.131 | -0.68 (-1.79, 0.43) | 0.228 | -0.60 (-1.69, 0.49) | 0.280 |
| Busy traffic | All of the time | Reference |  | Reference |  | Reference |  |
|  | Most of the time | 0.07 (-0.34, 0.48) | 0.743 | 0.98 (0.20, 1.77) | **0.014** | -0.06 (-0.62, 0.51) | 0.839 |
|  | Sometimes | 0.20 (-0.23, 0.63) | 0.359 | 0.93 (0.24, 1.63) | **0.009** | 0.02 (-0.66, 0.70) | 0.954 |
|  | Hardly ever/Never | 0.71 (0.15, 1.27) | **0.013** | 1.89 (0.79, 2.98) | **0.001** | 0.31 (-0.63, 1.24) | 0.520 |
| Parked cars | All of the time | Reference |  | Reference |  | Reference |  |
|  | Most of the time | -0.11 (-0.43, 0.21) | 0.506 | -0.22 (-0.64, 0.20) | 0.301 | -0.13 (-0.80, 0.54) | 0.697 |
|  | Sometimes | -0.30 (-0.66, 0.07) | 0.114 | -0.70 (-1.38, -0.02) | **0.045** | -0.12 (-0.70, 0.46) | 0.686 |
|  | Hardly ever/Never | -0.20 (-0.79, 0.39) | 0.502 | -1.17 (-1.89, -0.44) | **0.002** | 0.24 (-0.92, 1.40) | 0.685 |
| School decile | High | Reference |  | Reference |  | Reference |  |
|  | Medium | -0.33 (-1.00, 0.34) | 0.332 | -1.38 (-2.58, -0.18) | **0.024** | -1.28 (-2.66, 0.10) | 0.069 |
|  | Low | -0.06 (-0.99, 0.87) | 0.902 | -1.21 (-2.44, 0.02) | 0.054 | -1.42 (-2.82, -0.02) | **0.047** |
| Travelwise | No | Reference |  | Reference |  | Reference |  |
|  | Yes | 0.29 (-0.33, 0.91) | 0.354 | -0.70 (-1.62, 0.21) | 0.130 | -0.84 (-1.86, 0.18) | 0.104 |
| NDAI-C | - | 0.00 (-0.02, 0.02) | 0.878 | 0.00 (-0.03, 0.03) | 0.851 | -0.02 (-0.06, 0.02) | 0.327 |
| Cycle lane | - | -2.19 (-5.53, 1.14) | 0.197 | -4.18 (-7.36, -1.00) | **0.010** | 0.18 (-4.31, 4.67) | 0.937 |
| Traffic lights | - | 0.10 (-0.14, 0.34) | 0.408 | 0.36 (0.02, 0.70) | **0.041** | -0.51 (-0.76, -0.27) | **<0.001** |
| NDAI-C = child-specific neighbourhood destination accessibility index. **Bold**: *p* < 0.05. *Mixed effects multinomial logistic regression models (reference category = car). Model fit indices: Akaike Information Criterion (AIC) = 14868.7, Bayesian Information Criterion (BIC) = 14883.4. | | | | | | | |

Appendix E: Sensitivity analyses and comparison of model fit in the final model with that in models with different random intercept or variables (N = 1081)

|  | Random intercept | Variables excluded | Model fit indices | |
| --- | --- | --- | --- | --- |
|  |  |  | AIC | BIC |
| Final model (Model 3) | School | High & low traffic exposure, school walkability | 14868.7 | 14883.4 |
| Sensitivity analysis 1 | Neighbourhood | High & low traffic exposure, school walkability | 15147.9 | 15162.7 |
| Sensitivity analysis 2 | School | Low traffic exposure | 15058.3 | 15073.0 |
| N = number, AIC = Akaike Information Criterion, BIC = Bayesian Information Criterion. | | | | |
